# Changes in the anterior segment after cycloplegia with a biometer using swept-source optical coherence tomography

**DOI:** 10.1371/journal.pone.0183378

**Published:** 2017-08-14

**Authors:** Tomoaki Higashiyama, Maki Iwasa, Masahito Ohji

**Affiliations:** Department of Ophthalmology, Shiga University of Medical Science, Otsu, Japan; Massachusetts Eye & Ear Infirmary, Harvard Medical School, UNITED STATES

## Abstract

The aim of this study was to investigate changes in the anterior segment of the eye after cycloplegia. A biometer combined with swept-source optical coherence tomography (SSOCT) was used for measurements. Patients with strabismus or amblyopia who underwent cycloplegia were included. The axial length, central corneal thickness, anterior chamber depth, and lens thickness were measured with the biometer–SSOCT system before and after cycloplegia. Altogether, 10 eyes of 10 patients (mean age 7.20 ± 3.08 years, range 4–14 years) were evaluated. The mean measurements before cycloplegia were 22.75 ± 0.96 mm axial length, 516 ± 33 μm central corneal thickness, 3.40 ± 0.21 mm anterior chamber depth, and 3.77 ± 0.26 mm lens thickness. The corresponding values after cycloplegia were 22.75 ± 0.95 mm, 519 ± 34 μm, 3.68 ± 0.16 mm, and 3.42 ± 0.20 mm, respectively. The mean lens thickness had significantly decreased (*P* < 0.001) after cycloplegia, and the mean anterior chamber depth had significantly increased (*P* < 0.001). The means of the axial length (*P* = 0.66) and central corneal thickness (*P* = 0.17) had not changed significantly. The change in lens thickness was significantly correlated with the change in anterior chamber depth (*r* = −0.73, *P* = 0.02). The new biometer–SSOCT combination proved useful for accurately detecting changes in the anterior segment of the eye after cycloplegia in pediatric patients. The biometer’s measurements indicated increased anterior chamber depth and decreased lens thickness after cycloplegia. The anterior chamber depth increased relative to the decrease in lens thickness.

## Introduction

Refractive errors are related to amblyopia and strabismus in some pediatric patients. Cycloplegic agents are used routinely during the examination in pediatric patients to investigate the correct refraction without accommodation [1–6]. Because the ciliary muscle controls accommodation by changing the thickness of the lens, the cycloplegic agent causes paralysis of the ciliary muscle, thereby causing loss of accommodation. Cyclopentolate and atropine are the agents most commonly used to achieve cycloplegia [1, 4, 7]. Some previous studies with biometry using A-mode ultrasonography reported that the mean lens thickness was significantly decreased after cycloplegia [4, 8]. This measurement with a biometer and ultrasonography, however, is sometimes difficult and inaccurate in pediatric patients because the ultrasound device must come into contact with the cornea.

A new biometer using swept-source (SS) optical coherence tomography (OCT) was recently developed [9]. SSOCT is a Fourier-domain OCT, and the wavelength of the light source is turned in rapid cycles to scan the eye [10]. OCT improves the signal-to-noise ratio because the narrow-bandwidth wavelength light source improves the image quality and tissue penetration [10–12]. Thus, the biometer–SSOCT combination can accurately and speedily measure axial length, corneal thickness, anterior chamber depth, and lens thickness without contact [13–19].

We therefore investigated the following questions with this biometer–SSOCT combination. First, is biometry useful for measurements in pediatric patients? Second, how do the parameters of the eye's anterior segment change after cycloplegia? To answer these questions, we investigated the change in the anterior segment after cycloplegia with the biometer–SSOCT system.

## Methods

Patients with strabismus or amblyopia who underwent cycloplegia were included in this study that was conducted at the Department of Ophthalmology, Shiga University of Medical Science Hospital from April 2017 to May 2017. None of the patients had any other ocular disease.

This study was approved by the institutional review board of Shiga University of Medical Science, which was conducted in accordance with the tenets of the Declaration of Helsinki. The ethics committee stated that the patients' informed consent was not needed because the examinations were routinely performed, with the data extracted from the medical records and used in this study. In addition, the study design was displayed on the hospital website so if the participants had any objections they could notify us.

The axial length, central corneal thickness, anterior chamber depth, and lens thickness data were obtained with Argos (Suntec, Inc., Aichi, Japan), the biometer used in this study with SS-OCT. The instrument has an A-scan rate of 3000 lines/sec and is used as the light source centered on 1050 nm, which produces less scatter. The alignment was subsequently adjusted using an internal fixation lamp. The refraction data were obtained with the RKT-7700 system (NIDEK Co. Ltd., Aichi, Japan). The spherical equivalent was calculated by adding half of the cylindrical power to the spherical power.

Cycloplegia was performed using the following regimen http://dx.doi.org/10.17504/protocols.io.i5bcg2n.[PROTOCOL DOI]). Cyclopentolate hydrochloride 1% (Cyplegin 1% ophthalmic solution, Santen Pharmaceutical, Osaka, Japan) was instilled three times at 10-min intervals. The measurements were obtained 60 min after the last instillation. After cycloplegia was established, the lens thickness was routinely determined using the biometer, and the refraction was obtained with the autorefractometer to confirm the effects of cycloplegia because the cyclopentolate may be washed out due to some pediatric patients' crying. Although both eyes of each patient underwent the regimen, only the findings in the right eye of each patient were used in this study, regardless of which eye was affected by disease.

### Statistical analysis

The Shapiro–Wilk test was used to test the normality of the numerical variables. A paired *t*-test was used to compare the length of each tissue and the refraction before and after cycloplegia. We analyzed the correlations using Pearson's product–moment correlation coefficient. SPSS Statistics 22 software (IBM Corp., Armonk, NY, USA) was used for all analyses. Data are expressed as means ± standard deviation (SD). Values of *P* < 0.05 were considered to indicate statistical significance.

## Results

### Patients' characteristics

In all, 10 eyes of 10 patients (7 female, 3 male; mean age 7.20 ± 3.08 years, range 4–14 years) were studied. The diagnoses of the patients were anisometropic amblyopia in three cases, ametropic amblyopia in one case, esotropia in one case, and exophoria in five cases. The characteristics of the patients are shown in Table 1.

**Table 1:** Patients' characteristics.

Case	Age	Gender	Diagnosis
1	5	M	Exophoria
2	4	F	Exophoria
3	7	M	Esotropia
4	4	F	Ametropic amblyopia
5	9	F	Anisometropic amblyopia
6	6	F	Exophoria
7	6	F	Exophoria
8	10	M	Anisometropic amblyopia
9	14	F	Exophoria
10	7	F	Anisometropic amblyopia

F, female; M, male

### Anterior segment

**Fig 1:**
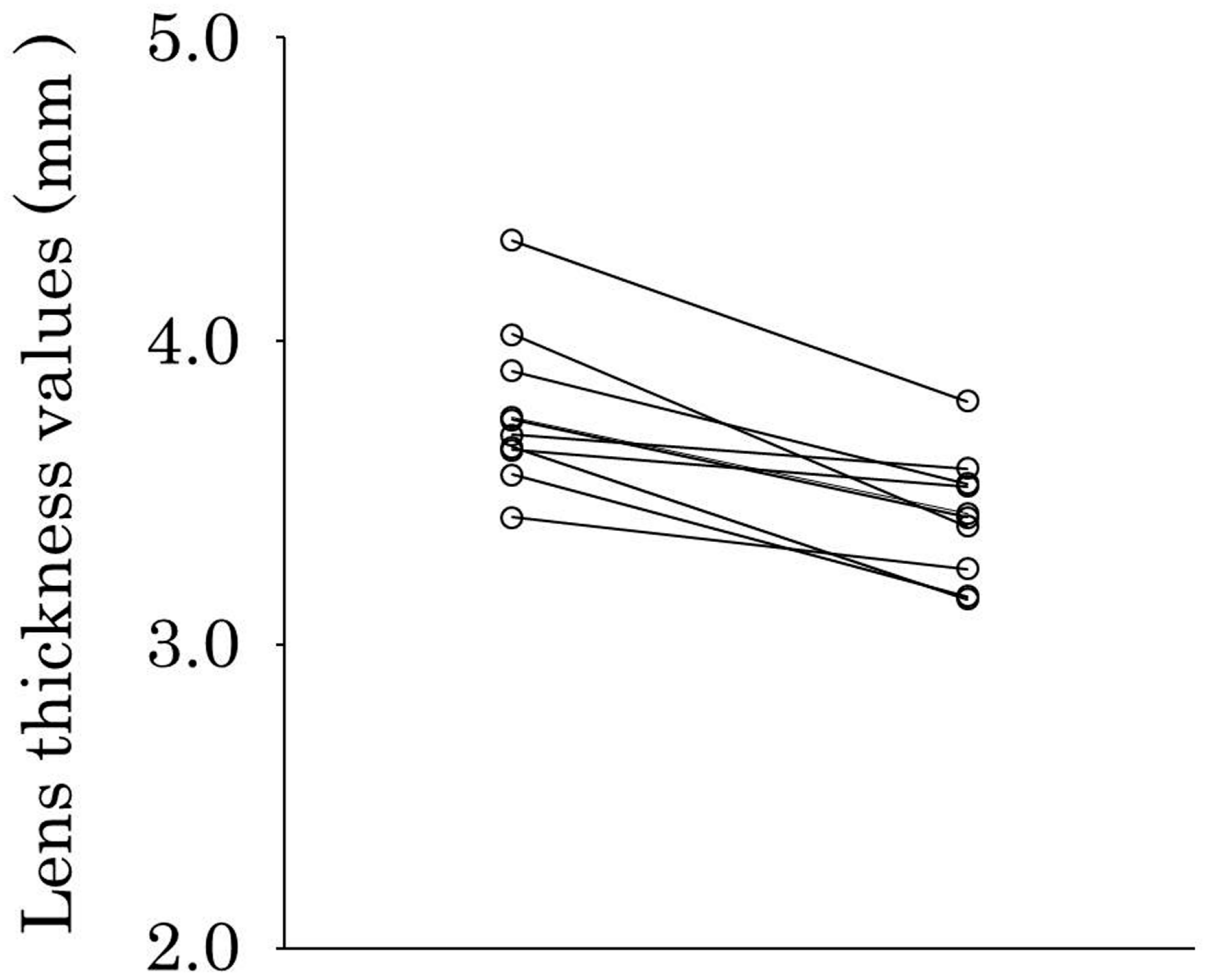
Lens thickness before and after cycloplegia. Left plots, before cycloplegia; right plots, after cycloplegia. Lens thickness values had decreased in all patients after cycloplegia, and the mean lens thickness had significantly decreased after cycloplegia (*P* < 0.001).

**Fig 2:**
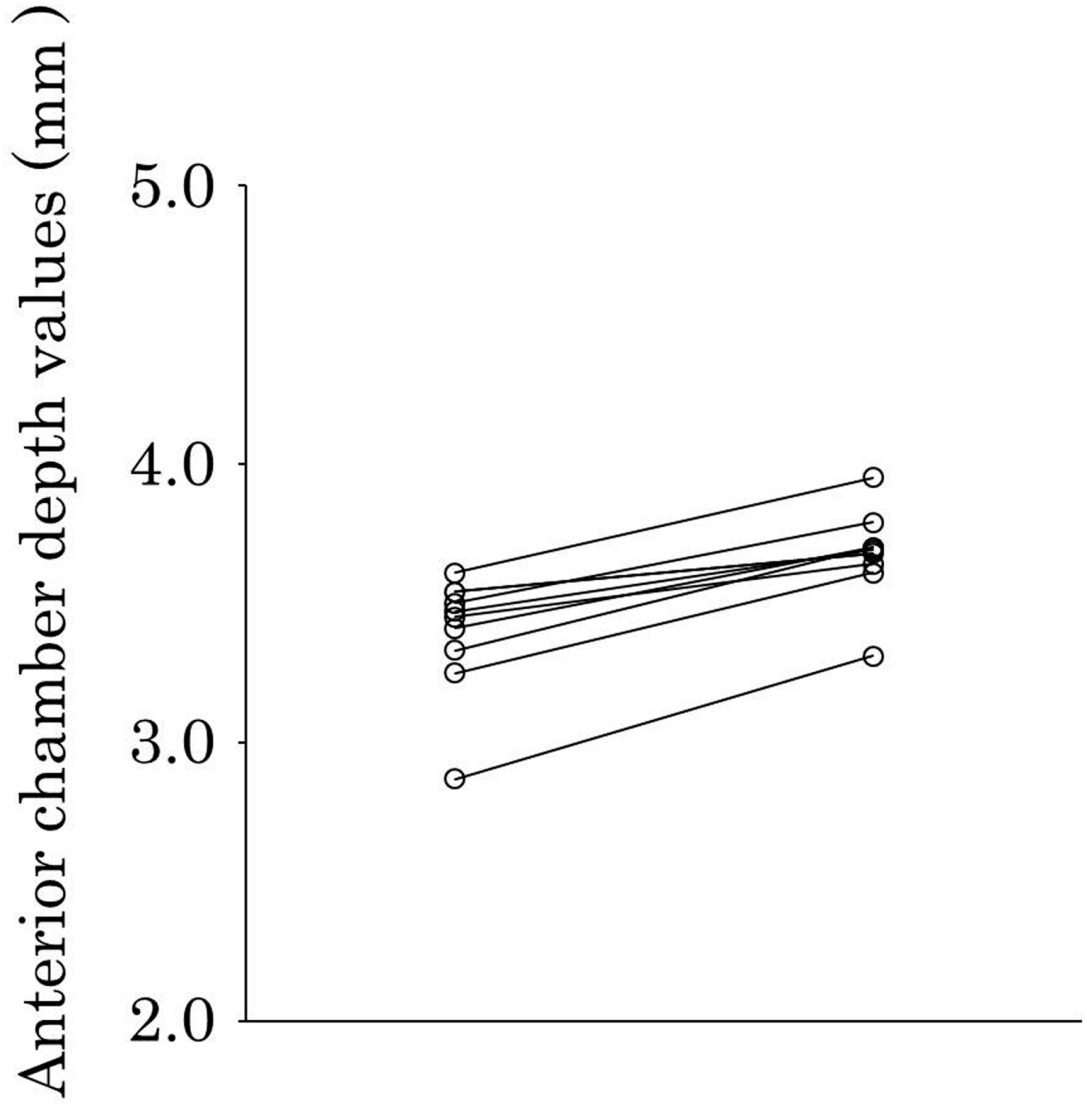
Anterior chamber depth before and after cycloplegia. Left plots, before cycloplegia; right plots, after cycloplegia). Anterior chamber depth values had increased in all patients after cycloplegia, and the mean anterior chamber depth had significantly increased after cycloplegia (*P* < 0.001).

The mean measurements before and after cycloplegia are shown in Table 2 and the measurement of each patient before and after cycloplegia are shown in Table 3. The lens thickness values had decreased in all patients, and the mean lens thickness had significantly decreased after cycloplegia (*P* < 0.001) (Fig 1). The anterior chamber depth values had increased in all patients, and the mean anterior chamber depth had significantly increased after cycloplegia (*P* < 0.001) (Fig 2). The mean axial length (*P* = 0.66) and mean central corneal thickness (*P* = 0.17) had not changed significantly after cycloplegia.

**Table 2:** Anterior segment and refraction before and after cycloplegia.

Parameter	Mean ± SD		P value
	Before	After	
Axial length (mm)	22.75 ± 0.96	22.75 ± 0.95	0.66
Central cornea thickness (μm)	516 ± 33	519 ± 34	0.17
Anterior chamber depth (mm)	3.40 ± 0.21	3.68 ± 0.16	<0.001
Lens thickness (mm)	3.77 ± 0.26	3.42 ± 0.20	<0.001
Spherical power (D)	0.10 ± 1.62	0.78 ± 1.46	0.04
Cylinder power (D)	-0.68 ± 0.53	-0.68 ± 0.54	1.00
Spherical equivalent (D)	-0.24 ± 1.64	0.44 ± 1.46	0.04

D, diopter.

**Table 3:** Anterior segment and refraction of each patient before and after cycloplegia.

	Case	1	2	3	4	5	6	7	8	9	10
Axial length (mm)	Before	23.62	22.84	24.26	21.72	22.40	22.41	21.96	23.58	23.44	21.28
	After	23.60	22.85	24.24	21.72	22.39	22.40	21.98	23.57	23.45	21.29
Central cornea thickness (μm)	Before	505	528	500	529	499	579	510	463	491	556
	After	508	536	520	538	497	574	507	462	490	565
Anterior chamber depth (mm)	Before	3.50	3.41	3.61	3.45	3.54	3.25	3.47	3.33	3.54	2.87
	After	3.79	3.70	3.95	3.64	3.68	3.61	3.69	3.70	3.68	3.31
Lens thickness (mm)	Before	3.75	4.02	3.74	3.64	3.42	3.65	3.56	3.90	3.69	4.33
	After	3.43	3.39	3.42	3.52	3.25	3.15	3.16	3.53	3.58	3.80
Spherical power (D)	Before	0.75	-2.50	-1.50	0.75	0.00	1.00	1.00	0.75	-2.00	2.75
	After	0.50	-0.50	0.00	2.25	0.00	2.50	1.25	1.25	-2.00	2.50
Cylinder power (D)	Before	-0.25	-0.75	-0.50	-2.00	-1.00	-0.75	-0.25	-0.25	-0.50	-0.50
	After	0.00	-1.00	-0.50	-1.75	-1.00	-1.00	0.00	-0.25	-0.75	-0.50
Spherical equivalent (D)	Before	0.63	-2.88	-1.75	-0.25	-0.50	0.63	0.88	0.63	-2.25	2.50
	After	0.50	-1.00	-0.25	1.38	-0.50	2.00	1.25	1.13	-2.38	2.25

D, diopter.

### Correlation between lens thickness and anterior chamber depth

**Fig 3:**
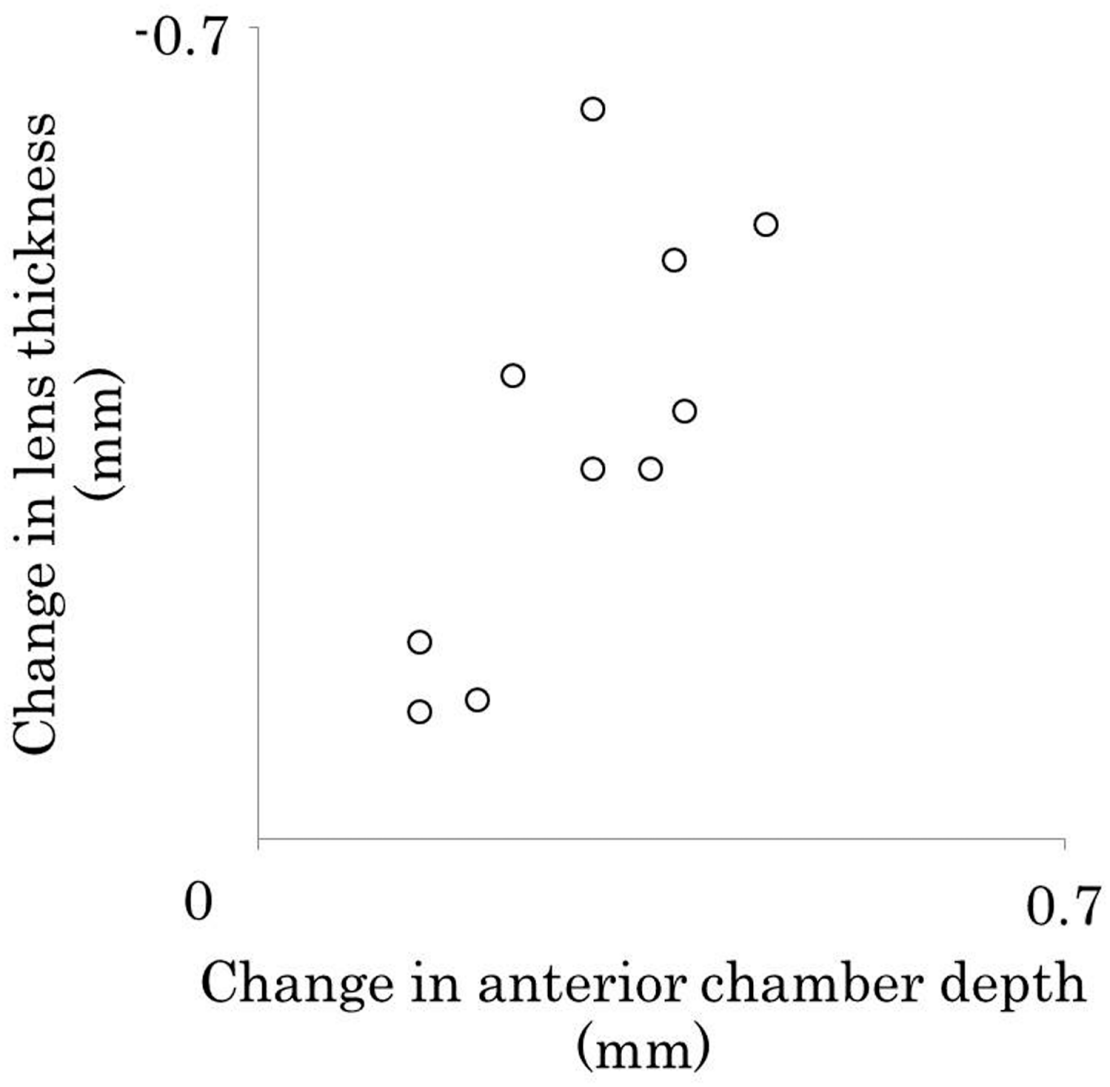
Correlation between changes in the lens thickness and those in the anterior chamber depth. The change in lens thickness was significantly correlated with the change in the anterior chamber depth (*r* = −0.73, *P* = 0.02).

The mean change in lens thickness was −0.35 ± 0.18 after cycloplegia, and the mean change for the anterior chamber depth was 0.28 ± 0.10. The change in lens thickness was significantly correlated with the change in anterior chamber depth (*r* = −0.73, *P* = 0.02) (Fig 3).

### Refraction

For the refraction parameters, the means before and after cycloplegia are shown in Table 2. Spherical power (*P* = 0.04) and spherical equivalent (*P* = 0.04) means had significantly increased after cycloplegia, whereas that of cylindrical power had not significantly changed (*P* = 1.00).

### Correlation between lens thickness and refraction

**Fig 4:**
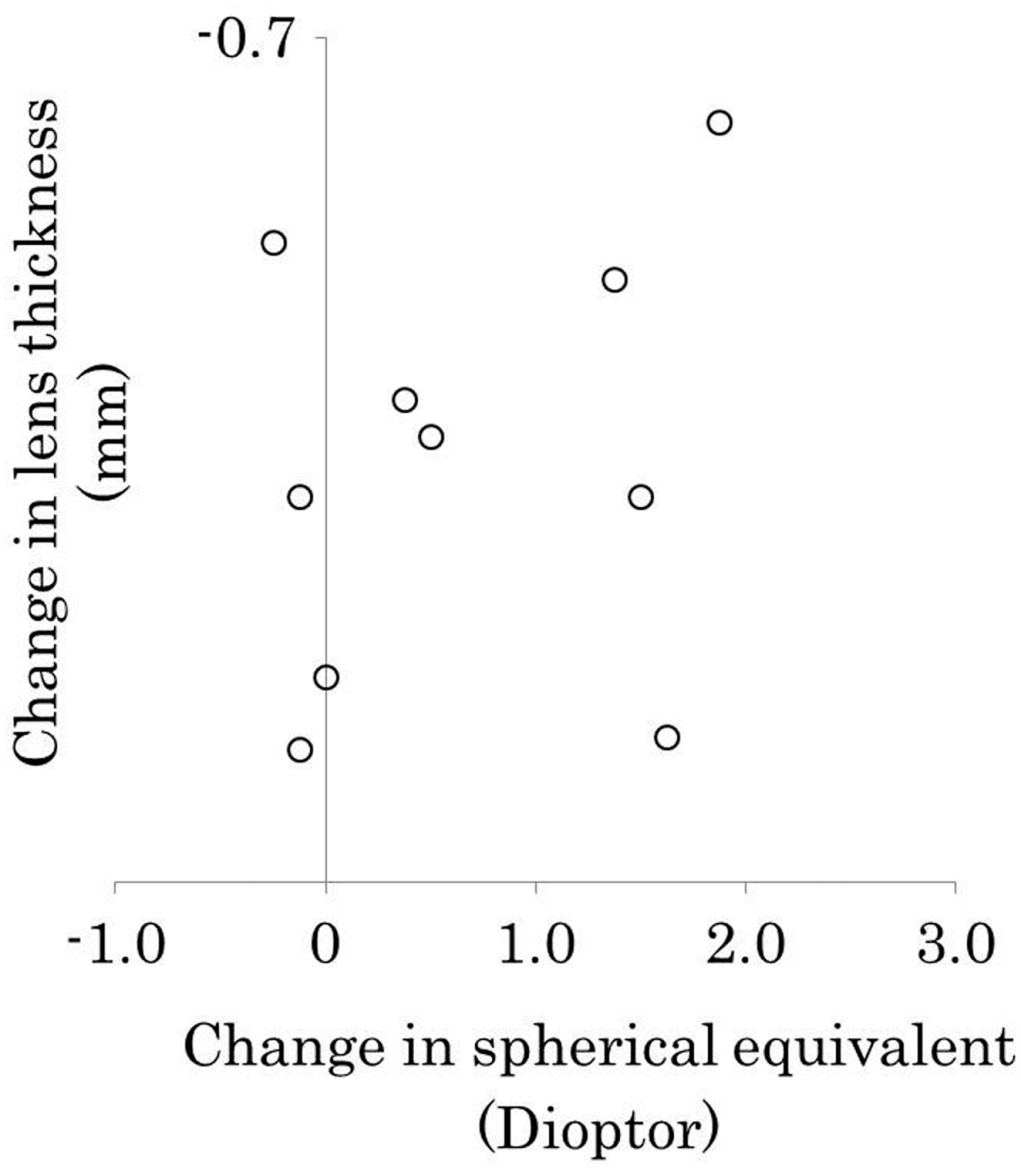
Correlation between lens thickness and refraction. The change in lens thickness was not significantly correlated with the change in the spherical equivalent (r = −0.27, *P* = 0.46).

The mean changes in lens thickness was −0.35 ± 0.18, and that in the spherical equivalent was 0.68 ± 0.83. Thus, the change in lens thickness was not significantly correlated with that of the spherical equivalent (*r* = −0.27, *P* = 0.46) (Fig 4).

## Discussion

This study provided three important clinical observations. First, measurements with non-contact biometry could be performed in pediatric patients. Second, the mean lens thickness had significantly decreased after cycloplegia, and that of the anterior chamber depth had significantly increased. Third, the change in lens thickness was significantly correlated with the change in the depth of the anterior chamber.

The measurements in this study were obtained using a biometer–SSOCT combination, whereas those in previous studies had used a biometer–ultrasonography combination [4, 5]. The SSOCT has three advantages over ultrasonography: accuracy, non-contact measurement, speed. As for accuracy, the repeatability and reproducibility of the SSOCT have been well shown [20]. In addition, the axial length and central corneal thickness did not significantly change in our study. In contrast, in a previous report using ultrasonography, changes in the axial length were not consistent. That is, the axial length increased in hyperopic eyes but decreased in myopic eyes [8]. Second, the biometer–SSOCT system allows non-contact measurements, as clearly shown in pediatric patients in the current study. The youngest patient in this study was 4 years of age, whereas the youngest patients in the previous study using the biometer–ultrasonography system were 5 and 7 years old [4, 5]. Shimizu et al. reported on the structure of the anterior chamber in children and adults with anterior segment OCT. The youngest patient in that study was 3 years old [21]. Thus, the non-contact biometer is useful for these measurements in pediatric patients. Third, measurements with the biometer–SSOCT combination can be performed speedily [20], which is an advantage in pediatric patients.

Our results showed that the change in lens thickness was significantly correlated with the change in anterior chamber depth. To the best of our knowledge, the relation between changes in lens thickness and the changes in the depth of the anterior chamber was not addressed in the previous study using the biometer–ultrasonography combination, although that study reported that lens thickness and vitreous cavity length were significantly decreased after cycloplegia and the anterior chamber depth was significantly increased [8]. Our study went further to reveal that the anterior chamber depth increased relative to the lens thickness decrease. Hence, although the deep anterior chamber must still be carefully examined for evidence of glaucoma in pediatric patients, based on our current findings we now recognize that the anterior chamber deepens according to the decrease in lens thickness after cycloplegia. This new understanding should be taken into account to help us avoid misdiagnoses.

The present study has several limitations. First, the sample size was small. Only 10 eyes of 10 patients were studied. The results, however, are considered reliable because they were consistent in all patients. Second, cyclopentolate was used to achieve cycloplegia. Although atropine is the most powerful cycloplegic agent [8], cyclopentolate is often used to achieve cycloplegia in daily practice because its duration of action is shorter than that of atropine. Third, the change in lens thickness did not significantly correlate with that of the spherical equivalent. The reason might be that the measurement instruments were different.

In conclusion, the biometer–SSOCT combination is useful for accurately detecting changes in the anterior segment of the eye after cycloplegia in pediatric patients. This biometer showed the increased anterior chamber depth and the decreased lens thickness after cycloplegia. The anterior chamber depth was increased relative to the decrease in lens thickness.

## Supporting information

S1 Table Specific dataset for all individuals. (XLSX)
